# The Pregnancy and EARly Life study (PEARL) - a longitudinal study to understand how gut microbes contribute to maintaining health during pregnancy and early life

**DOI:** 10.1186/s12887-021-02835-5

**Published:** 2021-08-24

**Authors:** Sarah Phillips, Rachel Watt, Thomas Atkinson, George M Savva, Antonietta Hayhoe, Lindsay J Hall, on behalf of the PEARL study team, Lindsay J Hall, Sarah Philips, Rachel Watt, Martin Cameron, Paul Clarke, Jon Lartey, Antonietta Hayhoe, George Savva, Thomas Atkinson, Melissa Cambell-Kelly, Laura Harris, Hayley Summerfield

**Affiliations:** 1 Gut Microbes and Health, Quadram Institute Bioscience, Norwich Research Park, Norwich, NR4 7UQ UK; 2 Norwich Medical School, University of East Anglia, Norwich Research Park, Norwich, NR4 7TJ UK; 3 Intestinal Microbiome, ZIEL - Institute for Food & Health, School of Life Sciences, Technical University of Munich, Freising, Germany

**Keywords:** Microbiome, Pregnancy, Early-life, Gut health, Health

## Abstract

**Background:**

The early life period represents the first step in establishing a beneficial microbial ecosystem, which in turn affects both short and longer-term health. Changes during pregnancy influence the neonatal microbiome; through transmission of maternal microbes during childbirth, and beyond, through nutritional programming. However, in-depth exploration of longitudinal maternal-infant cohorts, with sampling of multiple body sites, complemented by clinical and nutritional metadata, and use of cutting-edge experimental systems are limited.

The PEARL study will increase our knowledge of; how microbes (including viruses/phages, bacteria, fungi and archaea) change in composition and functional capacity during pregnancy; transmission pathways from mother to infant; the impact of various factors on microbial communities across pregnancy and early life (e.g. diet), and how these microbes interact with other microbes and modulate host processes, including links to disease onset.

**Methods:**

PEARL is a longitudinal observational prospective study of 250 pregnant women and their newborns, with stool and blood samples, questionnaires and routine clinical data collected during pregnancy, labour, birth and up to 24 months post birth.

Metagenomic sequencing of samples will be used to define microbiome profiles, and allow for genus, species and strain-level taxonomic identification and corresponding functional analysis. A subset of samples will be analysed for host (immune/metabolite) molecules to identify factors that alter the host gut environment. Culturing will be used to identify new strains of health-promoting bacteria, and potential pathogens. Various *in vitro* and *in vivo* experiments will probe underlying mechanisms governing microbe-microbe and microbe-host interactions.

**Discussion:**

Longitudinal studies, like PEARL, are critical if we are to define biomarkers, determine mechanisms underlying microbiome profiles in health and disease, and develop new diet- and microbe-based therapies to be tested in future studies and clinical trials.

**Trial registration:**

This study is registered in the ClinicalTrials.gov Database with ID: NCT03916874.

**Supplementary Information:**

The online version contains supplementary material available at 10.1186/s12887-021-02835-5.

## Background

**Fig. 1:**
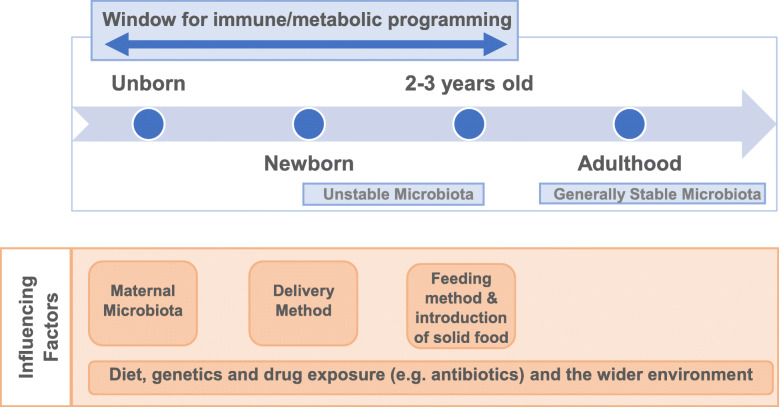
Overview of development of the microbiome during early life and the factors that influence this

The human gut microbiota comprises a complex microbial ecosystem (including viruses/phages, bacteria, fungi and archaea) which benefit their host through acquisition of additional nutrients and energy from dietary components, optimised development of the immune system, and resistance against pathogens [1, 2]. As the neonatal gut is essentially sterile, these beneficial microbes and their associated functions, must be acquired during and after birth; this is achieved through initial colonisation by pioneer bacteria, successive diversification, and changes in microbial population densities over time, until a climax or ‘stable’ microbiome is established during infancy and early childhood (Fig. 1) [3, 4].

The initial colonisation and establishment of the gut microbiome during early life influences physiological and immune development [4]. Critically, disturbances within this pioneering microbial community (both in mother and infant) have the potential to increase the risk of developing diseases such as autoimmune conditions, allergic-type disorders, infections and chronic intestinal diseases [4–6]. Factors such as antibiotic usage, diet (i.e. breast versus formula milk) and birth mode (i.e. Caesarean section versus vaginal) can affect the gut microbiota during this time (Fig. 1) [7–9].

Transmission of microbial species from the maternal microbiome to the infant occurs at birth, with subsequent waves of colonisation occurring as the infant ages [10–12]. There is also an increasing awareness of the importance of maternal health during pregnancy for infant development and health, both before and after birth [13]. Importantly, these life stage events appear to be governed by particular interactions (immune and dietary) that ‘select’ beneficial microbes such as *Bifidobacterium* [4, 14, 15]. Species in this genus are prevalent in the gastrointestinal tract of mothers in the later stages of pregnancy and can represent up to 80 % of the total microbiome in healthy infants [14, 16, 17]. These pioneering microbial species and strains contribute to ecosystem structuring, which is heavily influenced by their ability to metabolise complex sugars presents in breast milk – human milk oligosaccharides [17].

Although several studies have looked at these key factors at these critical timepoints, there are still many unknowns with respect to this key developmental window including; how the microbiome changes in response to different phases of pregnancy across different body sites; whether microbes from mothers are directly passed to infants during birth, and how birth mode affects this (i.e. vaginal birth vs. C-section); how factors like diet and antibiotics influence the maternal microbiome, and what impact this has on developing infant microbial communities; how different feeding regiments (e.g. breast vs. formula milk) influence specific microbial populations in the infant (e.g. *Bifidobacterium*); how these microbes influence immune and metabolic health.; and if in-depth mechanistic studies using *in vitro* and *in vivo* models can determine how specific microbes and communities contribute to healthy development and prevent disease incidence.

Longitudinal pregnancy and infant studies like PEARL, will provide an invaluable resource to study the importance of the early life microbiome. Gathering these data is critical for identification and development of new therapies and health- associated practices to improve health, both in the short- term and across the life course.

### Objectives

The primary objective of the PEARL study is to describe the determinants, function and composition of maternal and neonatal microbiomes throughout pregnancy and early childhood in a cohort of mothers and babies without serious health conditions.

Other objectives of this study are;

1. to link or correlate microbiome profiles to routinely collected clinical information in mothers and infants (e.g. antibiotic usage, birth mode) and dietary information via questionnaires (e.g. breast vs. formula milk), and to host metabolites and immune markers (e.g. cytokines) measured in samples collected;
2. to use microbiome samples in preclinical models to describe the role that the early- life microbiome plays in immune and metabolic development and resistance to diseases;
3. to use analysis and microbiome samples (using *in vitro* and preclinical models) to characterise the cause, effect and consequences of disturbances in the early-life microbiome due to external factors (e.g. antibiotics, birthing method, diet) on subsequent health outcomes;
4. to define and characterise early-life associated microbiome species (e.g. *Bifidobacterium* species and *Enterococcus* species) from samples for their probiotic/pathogenic traits (using *in vitro* and preclinical models).

An additional key output, which will be available to the wider research community, is a comprehensive collection of microbiome samples and associated clinical and diet/lifestyle information that will be accessible via requests to Professor Lindsay Hall and the Norwich Research Park (NRP) Biorepository, with all microbiome sequence data publicly available after deposition in public repositories.

## Methods

**Fig. 2:**
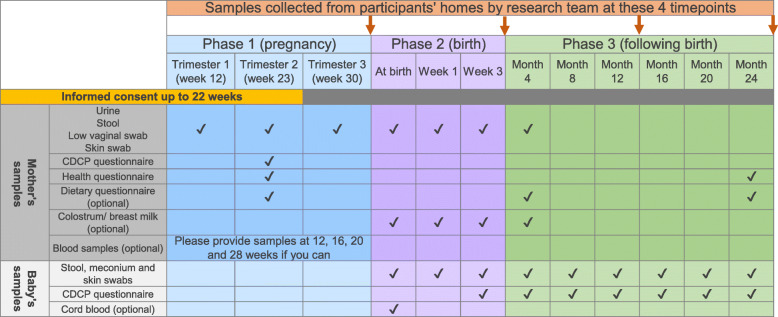
PEARL study overview

PEARL is a longitudinal study of 250 pregnant women and their new born(s). Biological samples and data will be collected in pregnancy, labour, birth and up to 24 months post birth. In the event of multiple live births, each infant will be included. Figure 2 provides an overview of the overall design of the study and shows which samples and data are collected at each timepoint. There are up to 12 collection timepoints, as well as 4 optional blood sample donations which will be collected at routine maternity care appointments. The study is divided into 3 collection phases: pregnancy, birth to 4 months, and 4 months to 24 months post birth.

Pregnant women can be recruited to PEARL at any time up to 22 weeks gestation. During phase 1 participants will be asked to collect samples and complete questionnaires during each of the three trimesters of pregnancy if they are involved in the study at that point. Participants are also able to donate blood samples at weeks 12, 16, 20, 28.

During phase 2, participants will collect samples at three timepoints from themselves and their infant(s); birth, at 1 week post birth, and at 3 weeks post birth.

Phase 3 collections will be every 4 months (4, 8, 12, 16, 20 and 24 months). With the exception of month 4, further collections of samples will be from the infant only.

At each collection time point, there is a window of 7 days within which the participant can collect their samples. This is to provide as much flexibility for participants as possible. Participants will receive a reminder message via text message or email when their samples are due to be taken, and again towards the end of the timepoint window.

Most samples will be collected at home using packs provided to the participants. The participants will be provided with a small freezer where they will store their samples until collected by a member of the research team. This freezer will be dedicated to holding study samples to minimize any cross-contamination risks to the participant. Collections from the participants’ homes will occur at 4 points throughout the study after phase 1, after phase two and at month 12 and month 24 during phase 3.

At birth, or any time the participant or her infant is an inpatient at the Norfolk and Norwich University Hospitals NHS Foundation Trust (NNUH), participants can collect their samples at the NNUH and give them to their midwife/obstetrician who will put these in the PEARL study freezer located at the hospital.

The sample collection protocol has been designed using significant optimisation information from our previous study, BAMBI [18], with additional information gathered from other longitudinal cohort studies including the Flemish Gut Project [19], and we are confident that our current protocol will allow participants to easily collect and store samples at -20◦C until collection.

After collection from participants’ homes, all samples will be appropriately aliquoted and will be stored in QIB at -80 °C prior to analysis. Additional aliquots may also be stored at the NRP Biorepository at -80 °C for future research if the participant has provided this additional consent.

Participants will complete 3 questionnaires during the study (timepoints detailed in Fig. 2) including questions on health, lifestyle, and diet. The questionnaires include:

- Centre for Disease Control and Prevention (CDCP) questionnaires for pregnancy, breastfeeding and infant feeding practices [20]. Modified, with permission, for the purposes of this study (supplementary files 1, 2, 3, 4, 5, 6 and 7).
- A health questionnaire to provide information on lifestyle, diet and medication/medical history, developed for the study (supplementary files 8 and 9).
- A dietary perceptions and preferences questionnaire, developed for the study (supplementary files 10, 11 and 12).

The research team will also collect; clinical information from GP and hospital records that relate to any change in the participants or her infants’ medical history during the course of the study; routine data collected by health visitors (developmental Ages & Stages data) which includes information on fine and gross motor skills and problem solving; and routine pregnancy scans and tests.

### COVID19 mitigation strategies

In response to the COVID-19 pandemic, it is important to implement a risk mitigation strategy to protect the health and safety of both participants and researchers. Participants will be advised that they should not collect samples if they or anyone in their household has COVID19 symptoms, are self-isolating, are awaiting test results, or have in the last 70 days tested positive for COVID19. The research team will send collection packs and arrange the collection of participants’ samples following Standard Operating Procedures (SOPs)/ COVID-19 risk-assessments.

### Participant selection

#### Sample size

PEARL will recruit 250 pregnant women. Our primary aim is to detect associations between aspects of neonatal and maternal microbiomes, and their associations with external factors. As an indicative power calculation, if a potential risk factor has a prevalence of 25 % (recent data suggests that 25 % of mothers who deliver at our recruitment hospital [Norfolk and Norwich University Hospitals NHS Foundation Trust – NNUH] do not initiate breast feeding; the UK Caesarean section rate is also currently around 25 %). Then with 200 observations we would have power of 80 % to detect a difference of 0.46 standard deviations in any given outcome measure between those with and without the feature at a *p*-value of 0.05. This is commonly understood to correspond to a ‘moderate’ size of effect between an exposure and an outcome.

For continuous outcomes or correlations between 250 mother and baby pairs, we would be able to detect correlations of *r* = 0.2 with 80 % at *p* < 0.05. This corresponds to a continuous feature accounting for r2 = 4 % of the population variation in any given characteristic of the microbiome.

For secondary analyses, if the *p*-value threshold for statistical significance is corrected to *p* < 0.005 to account for multiplicity then the corresponding smallest detectable differences are 0.6 standard deviations between groups defined by a binary factor (e.g. mode of delivery or breastfeeding) or correlation of 0.25 between continuous variables.

Previously, Azad et al. [21] explore effects of maternal antibiotic use, mode of delivery and breastfeeding on diversity and major taxon abundance among a cohort of 198 Canadian neonates at 3 and 12 months of age. Differences in the three most abundant taxa between elective Caesarean section (*n* = 19) and vaginal births (*n* = 96) were detected at 3 months with *p*-values of < 0.001 and < 0.01, corresponding to effect sizes of > 0.63 and > 0.49 standard deviations.

#### Cohort

Two hundred fifty healthy pregnant women will be recruited from across Norfolk. We will be recruiting participants from the maternity unit at a single major regional hospital, the Norwich and Norfolk University Hospital Foundation Trust (NNUH) which provides care for a population of approximately 1,000,000. The vast majority of women in the region will use the maternity services of this hospital. Participants will all be planning to give birth at NNUH or have a home birth with their maternity care provided by the NNUH. The total number of live births per year at NNUH is ~ 6000. Our primary objective is to describe the determinants, function and composition of maternal and neonatal microbiomes throughout pregnancy and early childhood in a cohort of mothers and their babies without serious health conditions. Therefore, we will be seeking a ‘healthy’ cohort of mothers, with the following inclusion and exclusion criteria applied.

##### Inclusion criteria

- Confirmed pregnancy and aged at least 18 years.
- Must be able to understand the requirements of the study and provide signed and dated informed consent for herself and her unborn child.
- Planning to give birth at NNUH or at home under the care of NNUH.
- At the point of study consent, be ≤ 22 weeks pregnant.
- Body Mass Index (BMI) between 18 and 35 kg/m2.
- Must be willing to provide biological samples over a period of 31 months (urine samples, stool samples, low vaginal swabs, skin swabs and breast milk (if breastfeeding this is optional). Blood samples are optional.
- Must be willing to accommodate a small freezer to store frozen samples for the duration of the study.
- Must be willing to complete study questionnaires over a period of 31 months.

##### Exclusion criteria

The participant may not enter the study if ANY of the following apply:

- Pregnancy is for surrogacy purposes.
- Planned adoption, fostering of baby or baby not planned to be living with biological mother.
- Currently taking part in an interventional study.
- Living with or related to a member of the research team.
- Current smoker.
- Taken antibiotics or antifungals or antivirals within the last 3 months.
- Taken steroids within the last 6 months.
- Currently taking more than a daily dose of probiotics.
- History of polyps within the gut.
- Long-standing gastrointestinal or liver function abnormality requiring on-going medical management or medication.
- Current or history of cancer except for squamous or basal cell carcinomas of the skin that have been medically managed by local excision.
- Unstable dietary history as defined by major changes in diet during the previous month, where the participant has stopped or significantly increased a major food group in the diet, for example changed to vegan, vegetarian or stopped eating red meat.
- History of alcohol, drug or substance abuse.
- History of Hepatitis B or Hepatitis C.
- Any confirmed or suspected pre-existing condition/state of immunosuppression or immunodeficiency (primary or acquired), for example Rheumatoid Arthritis, Type 1 Diabetes, Multiple Sclerosis, Asthma, Eczema and Psoriasis. (Participants who are asymptomatic of Asthma, Eczema and Psoriasis in the last 5 years can be included in the study).
- Major surgery of the gastrointestinal tract, apart from gall bladder or appendix removal, in the past 5 years.
- Any major bowel resection at any time.
- History of Ulcerative Colitis or Crohn’s Disease or Diverticulitis.
- Persistent, infectious gastroenteritis, gastritis, persistent or chronic diarrhoea of unknown cause, Clostridium difficile infection (recurrent) or Helicobacter pylori infection (untreated).
- Chronic constipation.

### Recruitment

Recruitment began in May 2019 and will continue until May 2022. Recruitment will be mainly opportunistic at the NNUH NHS Foundation Trust antenatal clinics and midwife led community clinics in GP surgeries and health clinics.

In response to the COVID19 pandemic, digital appointments have been put in place for pregnant women that replace the community clinics in GP surgeries and health clinics. Due to the efficiencies that this brings, it is anticipated that these appointments will remain common practise even after measures put in place to control the pandemic have been lifted. We, therefore, also intend to include virtual recruitment as a routine recruitment method for this study.

Following the first appointment a pregnant woman has as part of her routine maternity care at NNUH, eligible, patients considered to have a low risk pregnancy will be contacted by a research midwife by telephone to introduce the study and ask if the participant is interested in taking part. If the participant is interested, they will be provided with the participant information sheet (PIS) via email and a video/telephone call will be arranged to continue the informed consent process if they so wish. Participants may also contact the study researcher directly by entering their interest into the expression of interest form on the PEARL study web page, by telephone, or via email. Participants may request a face to face consent appointment which will be held at the Quadram Institute Clinical Research Facility, however, this may not be possible if there are local restrictions in place to manage the COVID19 pandemic. Written informed consent will be obtained following Good Clinical Practice (GCP) guidelines by the research midwife or a member of the research study team.

The participant will then be assigned their unique PEARL study participant number which will provide pseudonymisation for the participant throughout the study.

It will be clearly stated that the participant is free to withdraw from the study at any time for any reason without prejudice to future care, without affecting their legal rights, and with no obligation to give the reason for withdrawal.

### Sample banking at the NRP biorepository

A key aspect of the PEARL study involves creating biobank of samples and data which can be used in future ethically approved research. To enable this, and following Informed Consent for study participation, all participants will be asked if they would be willing for their samples to be stored anonymously at the Norwich Research Park (NRP) Biorepository during and after the end of the study for long term storage. The NRP Biorepository is a tissue bank licensed by the UK’s Human Tissue Authority (HTA) with appropriate ethics approval which was granted by the NHS Health Research Authority (HRA), East of England-Cambridge East Research Ethics Committee (REC). Samples stored at NRP Biorepository will be used in future ethically approved research. Participants consenting to the long-term storage of their samples at the NRP Biorepository are given the option to provide explicit consent for their samples to potentially be used in future ethically approved animal, cloning, or commercial studies, however, these aspects are optional and not required for samples to be stored long term in the NRP Biorepository. Data will be recorded by returning a copy of the consent form to the Biorepository. The participant will be asked to read the current version of the NRP Biorepository Information Sheet and to sign this consent if they wish to participate in this aspect of the study. If the participant declines long term storage at the NRP Biorepository, all samples at the end of the study will be destroyed appropriately in accordance with the Human Tissue Act requirements.

### Statistics and analysis

#### Interim analyses

Interim reports will include the number of accruals to the study, the quality of the samples returned, the completeness and validity of the questionnaire data returned. The report will also detail any missing data and any participants who are lost to follow-up.

#### Analysis of samples

Samples will be subjected to microbiome analysis using high-throughput sequencing following standardised protocols validated and quality controlled from BAMBI SOPs, including: shotgun metagenomics for species-level taxonomic identification; functional profiling; and analysis of the gut-associated antimicrobial resistance ‘resistome’ using open access bioinformatics pipelines and new pipelines.

Bioinformatics pipelines are constantly being improved/updated, thus we will use the most appropriate ones available at time of analysis which may include e.g. MetaPhlAn 3 [22]. Downstream statistical analyses will also be performed by using various packages in R.

Stool samples will also be analysed for host (immune/metabolite) molecules to determine which factors (e.g. antibiotics, diet) alter the host gut environment, which will be carried out using standard lab protocols (e.g. multi-plex cytokine assays, and NMR metabolomics). Samples will also be cultured (single and complex [i.e. model colon systems]) to identify new strains of beneficial or ‘probiotic’ bacteria such as *Bifidobacterium* species, and also any potential pathogens, for use in additional *in vitro* and *in vivo* studies to answer specific questions about how early life microbes modulate host functions.

### Description of statistical methods

A main aim of this study is to establish a cohort and repository of samples for future research, hence there are many possible future analyses that may be done using the samples collected as a part of this study.

For primary analyses, the composition of the microbiome in each sample (as defined by participant, site and time point) will be described in terms of individual taxon abundance and overall measures of diversity. Several key aspects of composition of the maternal and neonatal microbiome will be identified as candidates for association with measures of health status based on review of previous studies and theoretical considerations.

The dynamics of microbiome development and transmission from mother to baby will be determined by describing and correlating the distribution of key measures of structure and composition between samples.

The effect of factors that might affect microbiome composition, such as breastfeeding initiation and mode of delivery, will be assessed using regression models.

For exploratory (hypothesis generating) analyses, multivariate methods such as partial least squares regression modelling will be used to explore internal correlation structure within each sample and test whether any set of microbiome characteristics identified correlates with a hypothesised clinical factor.

Throughout, careful attention will be paid to the possibility of false positive results occurring through multiplicity given the large number of hypotheses being tested and the large number of parameters to be set in bioinformatics and statistical techniques. This will be mitigated as far as possible for each hypothesis by clearly pre-specifying each individual analysis in a statistical analysis plan detailing the coding of exposures, outcomes and covariates and primary statistical methods to be employed including any subgroup analyses. These statistical analysis plans will be developed and will be pre-registered following data collection so that the distributions of key variables are known but before relevant analysis of microbiome and health outcome information is undertaken. Exploratory and secondary analyses will be clearly reported as such in all outputs, and results from all analyses will be published irrespective of whether findings are predominantly positive or negative.

### Study management plan

#### Trial management group

The Trial Management Group including the Chief Investigator (Prof Lindsay Hall), the study research team, NHS Principal Investigators and Clinical Research Network’s (CRN) Research Nurses will be responsible for the day-to-day management of the trial. They will monitor all aspects of the conduct and progress of the trial, ensure that the protocol is adhered to and take appropriate action to safeguard participants and the quality of the trial itself.

#### Trial management oversight group

Management of the study will be overseen by a Trial Management Oversight Group whose membership is made up of representatives from;

- Health, Safety, Environment and Quality Assurance.
- QIB Sponsor Representative.
- Gut Microbes and Health QIB Programme Manager.
- QIB Statistician.
- Information Technology Security Specialist.
- NRP Biorepository.
- Patient and Public Involvement.
- QIB Bioinformatician.

The Trial Management Oversight Group will be responsible for overseeing the running of the study. They will ensure the monitoring and facilitating the progress of the study, and ensure the study is delivered within the projected timelines. Recruitment targets, success of data collection, and any specific issues arising will be addressed.

### Data management plan

#### Description of the data

Our data collected for this study will be the following: -.

- Clinical data and measurements taken as part of routine care (Antenatal and Postnatal Data Collection Case Report Forms).
- Participant-reported general health and lifestyle data (Participant Trimester 2 and Participant 24 months Post Birth Health Questionnaires – Supplementary files 8 and 9) and Participant Dietary Preferences and Perceptions Questionnaire (Participant Trimester 2, 4 months post birth and 24 months post birth – supplementary files 10, 11 and 12).
- CDCP questionnaires, (participant-reported data, dietary intake and allergies data – supplementary files 1, 2, 3, 4, 5, 6 and 7).
- Biological Sample data (blood, stool, urine, colostrum/breast milk, cord blood, low vaginal swabs, skin swabs and meconium samples).
- Data collected from NNUH Integrated Clinical Environment (ICE) system (routine pregnancy results), other relevant databases within the Trust and hospital notes where it is relevant to the study.

All of the above collected data will be anonymised and stored on a secure IT (Information Technology) system which only the study research team will have access to.

A data monitoring committee is not needed because the trial is not testing a drug or device and any safety concerns associated with the trial have been reviewed by the ethics committee.

#### Specific management of samples

All biological samples and data collected as part of the study will be pseudonymised. Laboratory results will be maintained in a database and will be in file formats that can be shared internally. Sample management at QIB and the NRP biorepository will be enabled by use of a locally acquired Laboratory Information Management System. Only anonymised individual-level data will be shared within study team members (QIB and NHS investigators).

#### Data collection / generation

Data from medical records (hospital notes and GP records) will be extracted onto study-specific, ethically approved, forms and then uploaded into secure databases which has shared access with appropriately authorised research staff working on the project in compliance with International Good Clinical Practice (GCP) standards.

#### Data sharing and access

The research protocol is registered at ClinicalTrials.gov (NCT03916874). The study team will ensure full compliance with the standards required for deposition of information in any relevant public databases. Only anonymised individual-level datasets will be shared outside the team. Consent forms clearly state the data sharing procedures for data generated from this study.

All data will be managed, protected and shared in accordance with the requirements the Biotechnology and Biological Sciences Research Council (BBSRC) Data Sharing Policy.

Direct access will be granted to authorised representatives from the Sponsor and host institution for monitoring and/or audit of the study to ensure compliance with regulations.

### Ethical and regulatory considerations

#### Declaration of Helsinki and relevant regulations

The Investigator will ensure that this study is conducted in accordance with the principles of the Declaration of Helsinki. The proposed research will be conducted in accordance with the conditions and principles of the International Conference on Harmonisation Good Clinical Practice, and in compliance with national law. The research will meet the requirements of the new EU General Data Protection Regulation (GDPR), UK Data Protection Act 2018 and relevant sponsor’s policies.

#### Expenses and benefits

There are no planned in-person study visits for this study. However, if a participant requests a face to face consent appointment at the Quadram Institute Clinical Research Facility, provisions will be made to reimburse the participant for car parking, and travel expenses at 45p per mile. As a thank you, participants will be sent a £20 shopping voucher once the 24-month samples and data have been received. At the end of the study, the participant will also have the option of keeping the freezer if they wish.

It is not anticipated that any post-trial care will be required. If any participant is harmed whilst taking part in this clinical research study as a result of negligence on the part of a member of the study team, QIB holds liability insurance for such circumstances.

## Discussion

Only by conducting in-depth longitudinal studies of large cohorts of hosts (i.e. mothers and their babies in the case of PEARL) can we identify the factors that are responsible for shaping and sustaining the microbiome in health, or for causing disturbances in disease.

Longitudinal studies, like PEARL, are critical if we are to define biomarkers and develop new diet- and microbe-based therapies to promote health during pregnancy and early life in future studies and clinical trials [23]. Our pregnant mother and baby cohort will provide a unique collection of samples robustly linked to detailed clinical information and early-life diet information, which will be available to the wider research community, thus providing a significant UK-based resource.

A significantly novel aspect of PEARL, is to input clinical samples and data into experimental model systems to show how the microbiome modulates specific early life developmental programming, and to ‘collect’ novel early life associated microbiome species (e.g. *Bifidobacterium, Ruminococcus, Enterococcus*) that can be used in these systems, and why may form the basis of for future microbiome modulation clinical trials.

Our protocol for PEARL from the first trimester of pregnancy and foetal development through to early childhood has been designed to complement other UK-based cohort studies looking at pregnancy and early life such as the BabyBiome Study [9] and COCO90s [24]. However, to the best of our knowledge, no study has attempted to undertake a comprehensive microbiome analysis throughout pregnancy and into infanthood, and none have linked this to detailed patient information that we are gathering through routine clinical data collection, lifestyle, and dietary information.

The major unanticipated operational issue impacting the PEARL study during 2020–2021 is the COVID-19 pandemic. With this, came implementation of national and local restrictions to reduce transmission of the virus. This included stay at home orders, social distancing and requirements to work from home where possible. The impacts on research studies such as PEARL have been significant.

Early in the pandemic, the PEARL study had to temporarily pause research activities while risk assessments could be urgently revised and adaptions to processes could be established to ensure safety of both participants and staff. We developed a remote process to recruit participants and introduced a reduced collection schedule from participants’ homes to reduce any face to face contact involved in taking part in the study. All sample collections adhere to local and national guidelines on COVID-19 restrictions set out by the government.

During a second wave of the pandemic and increased pressure on local NHS trusts, we had to temporarily pause all recruitment activity to release any NHS resource that we were using for recruitment activities.

We have seen an increased withdrawal rate to the study at times when local restrictions for COVID-19 have been implemented which could be attributed to the increased stress brought on by these ‘locked down’ environments, such as increased workload of working families to home school children. Therefore, not wishing to continue in the research environment.

We have made several amendments to the study to increase safety of participants and research staff during the pandemic, with the hope that these actions will mean the PEARL study will not be affected in the long term.

It is likely COVID-19 will have implications on future studies and risk assessments should be made prior to set up.

## Supplementary Information

**Additional file 1.** CDCP Participant Pregnancy Questionnaire Trimester 2.

**Additional file 2.** CDCP Newborn 3 Weeks Post Birth Questionnaire.

**Additional file 3.** CDCP Newborn 4 Months Post Birth Questionnaire.

**Additional file 4.** CDCP Newborn 8 Months Post Birth Questionnaire.

**Additional file 5.** CDCP Newborn 16 Months Post Birth Questionnaire.

**Additional file 6.** CDCP Newborn 20 Months Post Birth Questionnaire.

**Additional file 7.** CDCP Newborn 24 Months Post Birth Questionnaire.

**Additional file 8.** Participant Trimester 2 Health Questionnaire.

**Additional file 9.** Participant 24 Months Post Birth Health Questionnaire.

**Additional file 10.** T2 Participant Dietary Preferences and Perceptions Questionnaire.

**Additional file 11.** Four Months Post Birth Participant Dietary Preferences and Perceptions Questionnaire.

**Additional file 12.** Twenty-four Months Post Birth Dietary Preferences and Perceptions Questionnaire

## Data Availability

Data generated from the PEARL study will adhere to the Biotechnology and Biological Sciences Research Council (BBSRC) Data Sharing Policy. Anonymised datasets will be kept indefinitely and available to other researchers. Access to such data should be requested through the Chief Investigator, Professor Lindsay Hall. There will be no limitations to the dissemination of the results. It is anticipated that the results of this research project will be published and/or presented in a variety of forums, including peer reviewed journal publications, conference presentations, communications and media releases. In any publication and/or presentation, information will be provided in such a way that participants cannot be identified.

## Abbreviations

BAMBI study: Baby-Associated MicroBiota of the Intestine study
BBSRC: Biotechnology and Biological Sciences Research Council
BMI: Body mass index
CDCP: Centre for Disease Control and Prevention
CI: Chief investigator
CRN: Clinical Research Network
GCP: Good Clinical Practice (GCP)
GDPR: General Data Protection Regulation
GP: General Practitioner
HRA: Health Research Authority
HRGC: Human Research and Governance Committee
ICH GCP: International Conference on Harmonisation Good Clinical Practice
ICE: Integrated Clinical Environment
IT: Information Technology
NHS: National Health Service
NIHR: National Institute for Health Research
NNUH: Norfolk and Norwich University Hospitals NHS Foundation Trust
NRP: Norwich Research Park
PIS: Participant Information Sheet
QI CRF: Quadram Institute Clinical Research Facility
REC: Research Ethics Committee
SOPs: Standard operating procedures
