# Supplementary material for: The Pregnancy and EARly Life study (PEARL) - a longitudinal study to understand how gut microbes contribute to maintaining health during pregnancy and early life
Source: BMC Pediatr. 2021 Aug 24;21:357. doi: 10.1186/s12887-021-02835-5 (PMC8382937; doi:10.1186/s12887-021-02835-5)
Supplement: Supplementary file 1 — Additional file 1. CDCP Participant Pregnancy Questionnaire Trimester 2. [file 12887_2021_2835_MOESM1_ESM.pdf]

Participant Study ID ____________

The PEARL Study
Pregnancy and EARly Life

Participant Pregnancy Questionnaire Trimester 2 (Week 20 – 23)

This questionnaire has been modified from the approved Centre for Disease Control and Prevention (CDCP) questionnaire and has been designed to ask questions only relevant to this study for your convenience. CDCP have given their permission to use their Questionnaire for the purposes of this study.

SECTION A: HEALTH AND HEALTH CARE

1. When is your baby due? (PLEASE WRITE IN DAY AND MONTH)

DAY: ___________ MONTH: __________

2. What was your weight just before you became pregnant? __________ KGS

3. What is your current weight? _______ KGS. And what is your gestation point? ________ WEEKS

4. How tall are you? ____________ CM

5. What is your age? ____________ YEARS

6. Have you had gestational diabetes with this pregnancy? Yes .... ☐ No .... ☐ Don't know .... ☐

7. As best you know, which of the following health conditions do you yourself or your baby's other relatives have? (PLEASE 'X' ALL THAT APPLY)

- Juvenile onset diabetes (Type I) .... ☐
- Adult onset diabetes (Type II) .... ☐
- Asthma .... ☐
- Eczema .... ☐
- Food allergy .... ☐
- Allergies to pollen, dust, animals, latex, or anything else .... ☐
- Overweight or obesity .... ☐
- Any other chronic illness (please indicate which illness below) …… ☐

....

8. Have you taken any medication (e.g. antibiotics), including over-the-counter medication in the past 4 weeks? If so please list details below

....

9. Have you taken any supplements (e.g. probiotics, vitamin tablets) in the past 4 weeks? If so please list details below

....

THE NEXT QUESTIONS RELATE TO YOUR DIET

10. How many portions of milk and dairy foods (yogurt and cheese) do you eat or drink each day?*

- 1 portion a day ……………………………………………... ☐
- 2-3 portions a day ………………………………………… ☐
- 4-5 portions a day ………………………………………… ☐

*In answering this question please use the guidelines below.

1 portion = 200ml semi-skimmed milk

1 small pot yoghurt (125g)

30g-40g /1 oz cheese

11. How often do you eat processed meat or chicken products, e.g. meat pies, pastries, sausages, chicken nuggets each week?

- Every day ………………………………………………… ☐
- Every 5-6 days ………………………………………….. ☐
- Every 3-4 days ………………………………………… ☐
- Every 1-2 days ………………………………………… ☐
- Rarely ……………………………………………………… ☐

12. How many portions of fruit and vegetables (excluding potatoes) would you normally eat each day? *

- 0-2 portions a day …………………………………… ☐
- 3-4 portions a day …………………………………… ☐
- 5 or more portions a day ………………………….. ☐

*In answering this question please use the guidelines below.

1 portion = 1 slice of large fruit, e.g. melon, pineapple

1 medium sized fruit, e.g. a pear or a banana

2 small fruits, e.g. kiwis, mandarins or plums

1 cup of very small fruit, e.g. grapes or strawberries

1 glass of fruit juice (fruit juice can be counted as only one portion each day)

3 tablespoons of cooked vegetables

1 dessert bowl of salad

13. How many portions of bread, rice, potatoes, pasta and other starchy foods do you eat each day?*

- Less than 6 portions ……………………………….. ☐
- 6-9 portions …………………………………………….. ☐
- 10-12 portions ………………………………………….. ☐
- 12 or more portions ………………………………. ☐

*In answering this question please use the guidelines below.

1 portion = 1 slice of bread/toast

3 tablespoons of breakfast cereal

1 breakfast biscuit of Weetabix/ Shredded Wheat

1 medium sized boiled potato

½ medium baked potato

2 tablespoons of boiled rice

3 tablespoons of cooked pasta

14. How many portions of meat, fish or their alternatives, such as eggs, beans and other pulses, do you eat each day?*

- 1 portion a day or less ………………………………………. ☐
- 2 portions ………………………………………………………. ☐
- 3 or more portions …………………………………………… ☐

*In answering this question please use the guidelines below.

1 portion = 2 eggs

7 tablespoons cooked pulses, lentils, dahl or baked beans

115g quorn (equivalent to burger/ 2 sausages)

85g-115g/3-4oz chicken (if eaten)

115g-140g/4-5oz fish (equivalent to 1medium fillet white fish or a medium sized salmon steak)

Don't include processed meat products in your answer.

15. How often do you eat high fat, high sugar snacks in a week e.g. cakes, biscuits, crisps, chocolate bars, sweets?

- Every day ………………………………………………………… ☐
- 4-5 days a week ………………………………………………. ☐
- 2-3 days a week ………………………………………………. ☐
- Once a week …………………………………………………… ☐

16. How often would you eat a takeaway meal?

- Rarely ………………………………………………………………… ☐
- Once a fortnight ………………………………………………… ☐
- Once a week …………………………………………………… ☐
- 2-3 times a week ………………………………………………… ☐
- Every day ………………………………………………………… ☐

17. How many units of alcohol did you drink in the last 7 days (2 units = 1 glass of wine (175mL), or 3 units = 1 pint of beer)?

- 0 ………………………………………………………………………… ☐
- 2 ………………………………………………………………………… ☐
- 3-4 ……………………………………………………………………… ☐
- 5-8 ……………………………………………………………………… ☐
- More than 8 ……………………………………………………… ☐

18. Date you completed this form: Day _________ Month _________ Year __________

THANK YOU. Upon completion, please keep in a safe place and hand to designated staff when your frozen samples are collected.
