# Supplementary material for: The Pregnancy and EARly Life study (PEARL) - a longitudinal study to understand how gut microbes contribute to maintaining health during pregnancy and early life
Source: BMC Pediatr. 2021 Aug 24;21:357. doi: 10.1186/s12887-021-02835-5 (PMC8382937; doi:10.1186/s12887-021-02835-5)
Supplement: Supplementary file 2 — Additional file 2. CDCP Newborn 3 Weeks Post Birth Questionnaire. [file 12887_2021_2835_MOESM2_ESM.pdf]

Participant Study ID _______________

Baby Study ID ________________

The PEARL Study
Pregnancy and EARly Life

Newborn 3 Weeks Post Birth Questionnaire

This questionnaire has been modified from the approved Centre for Disease Control and Prevention (CDCP) questionnaire and has been designed to ask questions only relevant to this study for your convenience. CDCP have given their permission to use this modified version their Questionnaire for the purposes of this study and have agreed for Section C: Additional questions to be added.

SECTION A: YOUR NEW BABY'S BIRTH

1. Is your baby a boy or a girl? Boy .... ☐ Girl .... ☐

2. What was your baby's length at birth? ___________ CM

3. How was your baby delivered?

- Vaginally and not induced .... ☐
- A planned Caesarean .... ☐
- Vaginally and induced .... ☐
- An unplanned or emergency Caesarean .... ☐

4. Which of the following medications did you have during labour or delivery? (PLEASE 'X' ALL THAT APPLY)

- General anaesthesia (you were put to sleep) .... ☐
- Pudendal block/ other local blocks (injections into the vagina/ cervix before the birth) .... ☐
- A spinal or epidural .... ☐
- Other pain medication - you don't know which type .... ☐
- Nitrous oxide (gas breathed through a mask mouthpiece while remaining conscious) .... ☐
- No pain medication .... ☐

Participant Study ID ______________

Baby Study ID _______________

5. How much weight did you gain during this pregnancy? ___________ KGs

SECTION B: YOU AND YOUR BABY IN THE FIRST FEW WEEKS

6. How many nights were you in the hospital after your baby was born?

None ☐ 1 night ☐ 2 nights ☐ 3 nights ☐ 4 to 7 nights ☐ More than 7 nights ☐

7. In the past 7 days, how often was your baby fed each of the foods listed below? Include feedings by everyone who feeds the baby and include snacks and night-time feedings.

If your baby was fed the food once a day or more, write the number of feedings per day in the first column. If your baby was fed the food less than once a day, write the number of feedings per week in the second column.

Fill in only one column for each item. If your baby was not fed the food at all during the past 7 days, write 0 in the second column.

	FEEDINGS PER DAY	FEEDINGS PER WEEK
Breast milk	_______	_______
Formula (please write which type below)	_______	_______
....		
Water	_______	_______
Sugar water	_______	_______
Cow's milk or any other milk (rice, soy, goat, or other)	_______	_______
100% fruit or 100% vegetable juice	_______	_______
Sweet drinks (e.g. juice drinks, soft drinks, soda, sweet tea etc.)	_______	_______
Baby cereal	_______	_______
Other (PLEASE SPECIFY)	_______	_______

Participant Study ID _______________

Baby Study ID ________________

8. Was your baby given any medication (e.g. antibiotics), including over-the-counter medication in the past 2 weeks? If so please list details below

……………………………………………………………………………………………

9. Was your baby given any supplements (e.g. probiotics, vitamin drops) in the past 2 weeks? If so please list details below

……………………………………………………………………………………………

10. Does your baby have any serious, long-term medical problems? No .... ☐

Yes .... ☐

(PLEASE EXPLAIN BRIEFLY) ……………………………………………………………

SECTION C: ADDITIONAL QUESTIONS

11. How long was your baby's first bath delayed post birth?

0-12 hrs ☐ 12-24 hrs ☐ 24-48 hrs ☐ More than 48 hrs ☐

12. Is your baby enrolled in a day-care/nursery environment outside your home?

Yes ☐ If yes, how many days per week _______ No ☐

13. Are there any pets currently living in the house?

Yes ☐ No ☐

14. Date you completed this form: Day __________ Month __________ Year ___________

THANK YOU. Upon completion, please keep in a safe place and hand to designated staff when your frozen samples are collected, unless you are completing this form online.
