# Supplementary material for: The Pregnancy and EARly Life study (PEARL) - a longitudinal study to understand how gut microbes contribute to maintaining health during pregnancy and early life
Source: BMC Pediatr. 2021 Aug 24;21:357. doi: 10.1186/s12887-021-02835-5 (PMC8382937; doi:10.1186/s12887-021-02835-5)
Supplement: Supplementary file 5 — Additional file 5. CDCP Newborn 16 Months Post Birth Questionnaire. [file 12887_2021_2835_MOESM5_ESM.pdf]

Participant Study ID ___________

Baby Study ID ____________

The PEARL Study
Pregnancy and EARly Life

Newborn 16 Months Post Birth Questionnaire

This questionnaire has been modified from the approved Centre for Disease Control and Prevention (CDCP) questionnaire and has been designed to ask questions only relevant to this study for your convenience. CDCP have given their permission to use this modified version their Questionnaire for the purposes of this study and have agreed for Section C: Additional questions to be added.

SECTION A: BABY'S FEEDING AND HEALTH

1. In the past 14 days, how often was your baby/ toddler fed each food listed below? Include feedings by everyone who feeds the baby and include snacks and night-time feedings.

If your baby/ toddler was fed the food once a day or more, write the number of feedings per day in the first column. If your baby/ toddler was fed the food less than once a day, write the number of feedings per week in the second column.

Fill in only one column for each item. If your baby/ toddler was not fed the food at all during the past 7 days, write 0 in the second column.

	FEEDINGS PER DAY	FEEDINGS PER WEEK
Breast milk	________________	________________
Formula (write which type of formula below)	________	________
....		
Cow's milk	________	________
Other milk: soy milk, rice milk, goat milk, etc	________	________
Other dairy foods: e.g. yoghurt, cheese, ice cream, pudding, etc.	________	________

Other soy foods: e.g. tofu, frozen soy desserts, etc	________	________
100% fruit or 100% vegetable juice	________	________
Sweet drinks: e.g. juice drinks, soft drinks, soda, sweet tea, etc.	________	________
Baby cereal	________	________
Other cereals & starches: e.g. breakfast cereals, teething biscuits, crackers, breads, pasta, rice	________	________
Fruit	________	________
Vegetables	________	________
French fries	________	________
Meat, chicken, combination dinners	________	________
Fish or shellfish	________	________
Peanut butter, other peanut foods, or nuts	________	________
Eggs	________	________
Sweet foods: e.g. sweets, cookies, cake, etc.	________	________
Other (Please specify below)	________	________

……………………………………………………………………………………

Section A-2 Health

2. Which of the following problems did your baby/ toddler have during the past 4 weeks? (PLEASE 'X' ALL THAT APPLY)

- Fever .... ☐
- Runny nose or cold .... ☐
- Diarrhoea .... ☐
- Throat infection ………………………….….. ☐
- Sinus infection ………………………………….. ☐
- Urinary tract infection ……………………… ☐

- Vomiting .... ☐
- Cough or wheeze .... ☐
- Ear infection .... ☐
- Asthma .... ☐
- Colic .... ☐
- Food allergy .... ☐
- Fussy or irritable .... ☐
- Eczema (atopic dermatitis) .... ☐
- Reflux .... ☐
- None of these .... ☐

3. Was your baby/ toddler given any medication (e.g. antibiotics), including over-the-counter medication in the past 4 weeks? If so please list details below

....

4. Was your baby/ toddler given any supplements (e.g. probiotics, vitamin drops) in the past 4 weeks? If so please list details below

....

5. How many stools (dirty nappies) does your baby/ toddler usually have in a 24-hour period? If less than one a day, how many days usually pass between stools?

___________ NUMBER OF STOOLS IN 24 HOURS **OR** ONE STOOL EVERY_____________DAYS

6. How would you describe your baby/ toddler's stool in the past 7 days? (PLEASE 'X' ALL THAT APPLY)

Hard .... ☐ Formed .... ☐ Soft .... ☐ Semi-watery .... ☐ Watery .... ☐

7. How much did your baby/toddler weigh the last time he or she was weighed at a doctor's visit?

________ KGs Don't know .... ☐

8. What was the date of that weight?________DAY_________MONTH Don't know .... ☐

Participant Study ID ___________

Baby Study ID ____________

9. How long was your baby/toddler the last time he or she was measured at a doctor's visit?

________ CM Don't know .... ☐

10. What was the date of that measurement?________MONTH_______DAY Don't know .... ☐

11. Has your baby/ toddler been hospitalised for any reason or has your baby been taken to a hospital for any outpatient procedure or surgery in the past 4 weeks?

Yes .... ☐ No .... ☐

12. How many nights was your baby/ toddler in the hospital for the most recent problem? (Write in 0 if your baby did not stay overnight.) ___________NIGHTS

13. How many teeth does your baby/ toddler have now? (Write in 0 if none.)

____________ NUMBER OF TEETH

SECTION C: FOOD ALLERGY SECTION

14. Has your baby/ toddler ever had problems caused by food, such as an allergic reaction, sensitivity, or intolerance?

Yes .... ☐ No .... ☐ (IF **NO** GO TO QUESTION 22).

15. What were the problems caused by (PLEASE 'X' ALL THAT APPLY)

- Food your baby/ toddler ate (including infant formula) .... ☐
- Food your baby/ toddler was exposed to through breast milk because of something you ate .... ☐

16. How old was your baby the first time he or she had a problem with food? (Include food your baby reacted to through breast milk.)

1 month or less .... ☐ 2 months .... ☐ 3 months .... ☐ 4 months .... ☐ 5 months .... ☐

6 months .... ☐ 7 months .... ☐ 8 months .... ☐ 9 months .... ☐ 10 months .... ☐ 11 months ...☐

12 months .... ☐ 13 months .... ☐ 14 months .... ☐ 15 months .... ☐ 16 months .... ☐

17. Did you take your baby/ toddler to a medical doctor because of these problems with food?

Yes .... ☐ No .... ☐

18. If your baby/ toddler was tested or examined for food allergy, what method was used? (PLEASE 'X' ALL THAT APPLY)

If your baby was not tested or examined for food allergy, 'X' here ☐ and go to Question 7.

- Parents' description of symptoms.. ☐
- A skin test .... ☐
- A blood test such as RAST, or CAP-RAST .... ☐
- An oesophageal or intestinal study ....
- Food elimination (withdrawal of the specific food to see if symptoms disappeared) .... ☐
- Food challenge (introduction of a specific food to see if symptoms reappeared) .... ☐
- Other (PLEASE SPECIFY below) .... ☐

....

19. Was your baby/ toddler diagnosed by a medical doctor as having an allergy to any food?

Yes .... ☐ No .... ☐

20. What symptoms of a problem with food has your baby/ toddler had? (PLEASE 'X' ALL THAT APPLY)

Congestion .... ☐	Gassiness or stomach cramps .... ☐	Runny nose .... ☐
Vomiting .... ☐	Asthma or wheezing .... ☐	Diarrhoea .... ☐
Trouble breathing .... ☐	Constipation .... ☐	Coughing .... ☐
Colic .... ☐	Swollen eyes and or lips .... ☐	Irritability .... ☐
Hives or welts .... ☐	Sleeplessness .... ☐	Flushing .... ☐
Blood in stool .... ☐	Skin rash or eczema .... ☐	Loss of consciousness .... ☐

21. Please indicate which foods caused a problem for your baby (PLEASE 'X' ALL THAT APPLY)

- Cows' milk or other dairy products
  (including infant formula made with cows' milk ....
- Soy milk or other soy food (including infant formula made with soy) ....
- Eggs ....
- Peanuts, peanut butter, or peanut oil ....
- Nuts (such as, almonds, pecans, walnuts) ....
- Sesame seed, tahini, or sesame seed oil ....
- Fish, shellfish, or other seafood ....
- Beef, chicken or turkey..
- Wheat, gluten, or wheat starch ....
- Other grain or cereal (such as oats, barley) ....
- Fruit or fruit juice ....
- Vegetables ....
- Other food (PLEASE SPECIFY below)

SECTION D: ADDITIONAL QUESTIONS

22. How often do you typically bathe (full body) your infant?

Daily Every other day A few times per week Once per week

Other: Please list___________________

23. What skin care products and how often do you use on your baby (full body, not including nappy area)?

<u>**Product**</u>	<u>**Time**</u>	<u>**Response**</u> (1 – 4)
**A,** Cleansers (washes/soap bars/liquid soaps)	**1,** Daily	A =
**B,** Moisturizers (lotions / creams / balms / oils)	**2,** Every Other Day	B =
**C,** Water cleansing only	**3,** A Few Times Per Week	C =
	**4,** Once a week	

24. Is your baby enrolled in a day-care/nursery environment outside your home?

Yes If yes, how many days per week ______ No

25. Are there any pets currently living in the house?

Yes ☐ No ☐

26. Date you completed this form: Day_________Month__________Year ___________

THANK YOU. Upon completion, please keep in a safe place and hand to designated staff when your frozen samples are collected, unless you are completing this form online.
