# Supplementary material for: The Pregnancy and EARly Life study (PEARL) - a longitudinal study to understand how gut microbes contribute to maintaining health during pregnancy and early life
Source: BMC Pediatr. 2021 Aug 24;21:357. doi: 10.1186/s12887-021-02835-5 (PMC8382937; doi:10.1186/s12887-021-02835-5)
Supplement: Supplementary file 9 — Additional file 9. Participant 24 Months Post Birth Health Questionnaire. [file 12887_2021_2835_MOESM9_ESM.pdf]

Participant 24 Months Post Birth Health Questionnaire – 11 pages.

Date of completion ………/……..../……… Participant Study Number _________________
DD MMM YYYY

You may decline to answer any question.
For many of the questions please indicate your answers by circling ONE of the options for each of the questions or by writing your answer in the space provided:

General Information

1.) What is your age now?

……… years

2.) Have you moved to a new house

a. Within the past month?

b. Within the past 3 months?

c. Within the past 6 months?

d. Within the past year?

e. I have not moved to a new house in over a year.

3.) When did you last travel outside the UK for more than 2 days?

a. Within the past month

b. Within the past 3 months

c. Within the past 6 months

d. Within the past year

4.) What is your housing arrangement?

a) Owner-occupier
b) Privately rented accommodation
c) Social housing / council housing
d) Shared community housing, residential or nursing home

5.) Do you live:

a. Alone?

b. With only your spouse or long-term partner?

c. With other family members?

d. With spouse or long-term partner and other family members?

6.) How many other people do you live with in total?

a. None

b. One

c. Two

d. Three

e. More than three

7.) Do you eat most of your meals in your place of residence?

a. Yes

b. No

8.) Do you own any pets that live indoors?

a. Yes

b. No

9.) Which is your dominant hand?

a. I am right handed

b. I am left handed

c. I can perform the same functions using either hand

General Diet Information

10.) What type of diet do you usually eat?

a. Eat both plants and meats

b. Eat meat but not red meat

c. Vegetarian

d. Vegetarian but eat seafood

e. Vegan (eat no food from animals)

	DAILY	WEEKLY	MONTHLY	LESS THAN MONTHLY
11.) Do you take a multivitamin? If so, please tick when	☐	☐	☐	☐

12.) Are you regularly taking any other nutritional or herbal supplements?

if yes please indicate how often and what you are taking in the space below

	☐	☐	☐	☐

13.) Do you consume any probiotic based food products such as live yoghurts?

a. Yes, if yes *please state in the space below the number you consume in a week*

b. No, I do not consume any of these products

14.) Have you ever had lactose intolerance (diagnosed by a medical professional)?

a. Yes

b. No

15.) Do you have a diagnosis of gluten intolerance or diagnosed coeliac disease?

a. Yes – if so please specify?

b. No

c. Don’t know

16.) Have you ever had a diagnosed allergy to any of the following (please select all that apply)

a. Peanuts

b. Tree nuts, such as walnuts, brazil nuts….

c. Shellfish

d. Other (*please list below*)

e. I have no food allergies that I know of.

17.) Do you follow any other special diet restrictions other than those indicated above?

a. Yes, *if yes please explain in the space below*.

b. No

18.) Do you have any seasonal allergies, such as hay fever?

a. Yes

b. No

General Lifestyle and Hygiene Information

Exercise: Think about all the **moderate** activities that you did in the **last 7 days**. **Moderate** activities refer to activities that take moderate physical effort and make you breathe somewhat harder than normal. Think only about those physical activities that you did for at least 10 minutes at a time.

19. During the **last 7 days**, on how many days did you do **moderate** physical activities like carrying light loads, bicycling at a regular pace, or doubles tennis? Do not include walking or swimming.

_____ **days per week**

☐ No moderate physical activities → ***Skip to question 21***

20. How much time did you usually spend doing **moderate** physical activities on one of those days?

_____ **hours per day**

_____ **minutes per day**

☐ Don’t know/Not sure

Think about the time you spent **walking** in the **last 7 days**. This includes at work and at home, walking to travel from place to place, and any other walking that you might do solely for recreation, sport, exercise, or leisure.

21. During the **last 7 days**, on how many days did you **walk** for at least 10 minutes at a time?

_____ **days per week**

☐ No walking → ***Skip to question 23***

22. How much time did you usually spend **walking** on one of those days?

_____ **hours per day**

_____ **minutes per day**

☐ Don't know/Not sure

The last exercise question is about the time you spent **sitting** on weekdays during the **last 7 days**. Include time spent at work, at home, while doing course work and during leisure time. This may include time spent sitting at a desk, visiting friends, reading, or sitting or lying down to watch television.

23. During the **last 7 days**, how much time did you spend **sitting** on a **week day**?

_____ **hours per day**

_____ **minutes per day**

☐ Don't know/Not sure

24.) Do you bite your fingernails?

a. Yes

b. No

25.) How often do you use a public swimming pool or sauna?

a. Daily

b. Regularly (3-5 times/week)

c. Occasionally (1-2 times/week)

d. Rarely (few times/month)

e. Never

26.) How often do you smoke cigarettes?

a. Daily

b. Regularly (3-5 times/week)

c. Occasionally (1-2 times/week)

d. Rarely (few times/month)

e. Never

27.) Do you use a vaping/e-cigarette?

a. Yes

b. No

28.) How often do you drink alcohol?

a. Daily

b. Regularly (3-5 times/week)

c. Occasionally (1-2 times/week)

d. Rarely (few times/month)

e. Never

29.) How often do you brush your teeth?

a. Once a day

b. Twice a day

c. More than twice a day

d. Regularly (3-5 times/week)

e. Occasionally (1-2 times/week)

f. Rarely (few times/month)

g. Never

30.) Approximately how many hours of sleep to you get in an average night?

a. Less than 5 hours

b. 5-6 hours

c. 6-7 hours

d. 7-8 hours

e. 8 or more hours

General Health Information

31.) When did you last take antibiotics?

a. Within the past week

b. Within the past month

c. Within the past 3 months

d. Within the past year

e. I have not taken antibiotics in the past year.

32.) When did you last have a flu vaccine?

a. Within the past week

b. Within the past month

c. Within the past 3 months

d. Within the last year

e. I have not had the flu vaccine in the past year.

33.) Are you currently taking any prescribed or over the counter medications? (please select all that apply)?

a. To control blood pressure

b. To control cholesterol

c. For bowel symptoms such as constipation or diarrhoea/cramps

d. For any other short-lived disorders or conditions

e. For any other long-term disorders or conditions,

If yes for any of the above please PRINT the names of any drugs you are taking in the space below.

f. No I am not taking any prescribed or over the counter medication.

34.) Within the last 6 months, my weight has.

a. Increased more than 4 kilograms (9 pounds)

b. Decreased more than 4 kilograms (9 pounds)

c. Remained stable

d. Not sure

35.) Have you had your tonsils removed?

a. Yes

b. No

c. Don’t know

36.) Have you had your appendix removed?

a. Yes

b. No

c. Don’t know

37.) Have you had food poisoning?

a. If yes

Within the last 6 months?

Within the last year?

Within the last 2 years?

b. No

c. Don’t know

38.) Were you born via caesarean section (C-section)?

a. Yes

b. No

c. Don’t know

39.) As an infant were you breastfed?

a. Yes

b. No

c. Not sure

40.) Have you ever been diagnosed with Asthma? If yes, please indicate year diagnosed or circle unsure

a. Yes - Year diagnosed …………….. or unsure of year
b. Do you still suffer with Asthma now YES NO (Please circle)

b. No I have never had a diagnosis of Asthma

c. Not sure if I have ever had a diagnosis of Asthma

41.) Do you have diabetes? If Yes, please indicate year diagnosed or circle unsure

a. Yes, Type 1. Year diagnosed …………….. or unsure

b. Yes, Type 2. Year diagnosed …………….. or unsure

c. Yes but don’t know which Type. Year diagnosed …………….. or unsure

d. No I do not have diabetes

42.) How would you rate your health (circle your answer)

a. Excellent

b. Very Good

c. Good

d. Fair

e. Poor

43.) Using the Bristol Stool Chart below, please circle which type of stool you normally produce.

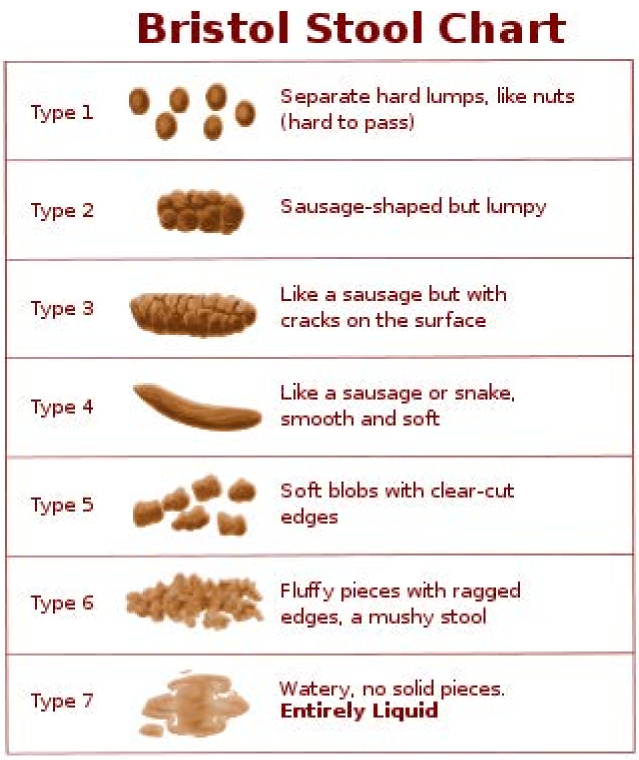

Thank you for completing this questionnaire.

If you have completed this as a paper version, please keep it in a safe place as it will be collected with your frozen samples.
