# Supplementary material for: The Pregnancy and EARly Life study (PEARL) - a longitudinal study to understand how gut microbes contribute to maintaining health during pregnancy and early life
Source: BMC Pediatr. 2021 Aug 24;21:357. doi: 10.1186/s12887-021-02835-5 (PMC8382937; doi:10.1186/s12887-021-02835-5)
Supplement: Supplementary file 12 — Additional file 12. Twenty-four Months Post Birth Dietary Preferences and Perceptions Questionnaire [file 12887_2021_2835_MOESM12_ESM.pdf]

University of East Anglia

Quadram Institute
Science Health Food Innovation

The PEARL Study
Pregnancy and EARly Life

*Participant Dietary Preferences and Perceptions Questionnaire 24 Months Post Birth. **Optional.***

Thank you for participating in this research. This is a questionnaire about your dietary preferences and how you understand your health needs during and after pregnancy.

When answering the questions, please remember that all information you provide will be anonymised.

Please answer the questions by putting a tick in the appropriate box.

Firstly, a few questions about you:

1. How many portions of fruit (fresh, tinned and frozen) did you eat yesterday? An apple, or an orange, a banana or a cupful of grapes counts as a portion ☐

2. How many portions of vegetables (fresh, tinned or frozen) did you eat yesterday? 80 grams or a cupful of vegetables counts as a portion. Beans, peas, tomatoes, seeds and nuts should be counted but not potatoes ☐

3. Would you say your fruit and vegetable consumption was?

Very low	a little low	about right	a little high	Very high
☐	☐	☐	☐	☐

4. Have you changed your fruit and vegetable consumption since you became pregnant?

Yes ☐ No ☐

5. Please tick one box that most reflects your thoughts.

For me eating 5 portions of fruit and vegetables per day is:

A). Not at all pleasant — Very pleasant

1 ☐ 2 ☐ 3 ☐ 4 ☐ 5 ☐

B). Not very convenient — Very convenient

1 ☐ 2 ☐ 3 ☐ 4 ☐ 5 ☐

C). Not at all enjoyable — Very enjoyable

1 ☐ 2 ☐ 3 ☐ 4 ☐ 5 ☐

D). Not in line with my food choice — In line with my food choice

1 ☐ 2 ☐ 3 ☐ 4 ☐ 5 ☐

E). Not at all important — Very important

1 ☐ 2 ☐ 3 ☐ 4 ☐ 5 ☐

6. For me eating 5 portions of fruit and vegetables per day is:

Very easy				Very difficult
1	2	3	4	5
☐	☐	☐	☐	☐

7. For me buying fruit and vegetables is:

Very cheap				Very expensive
1	2	3	4	5
☐	☐	☐	☐	☐

8. For me preparing and cooking fruit and vegetable is:

Very easy				Very difficult
1	2	3	4	5
☐	☐	☐	☐	☐

9. For me finding time to eat 5 a day is:

Very easy				Very difficult
1	2	3	4	5
☐	☐	☐	☐	☐

10. It is entirely my choice to eat 5 portions of fruit and vegetables per day

Strongly agree				Strongly disagree
1	2	3	4	5
☐	☐	☐	☐	☐

11. My attitude towards eating 5 portions of fruit and vegetables is:

Very positive				Very negative
1	2	3	4	5
☐	☐	☐	☐	☐

12. I think of myself as health conscious

Strongly agree				Strongly disagree
1	2	3	4	5
☐	☐	☐	☐	☐

13. I think of myself as someone who is concerned about the consequences of what I eat

Strongly agree				Strongly disagree
1	2	3	4	5
☐	☐	☐	☐	☐

14. My family think I should eat 5 portions of fruit and vegetables per day

Strongly agree				Strongly disagree
1	2	3	4	5
☐	☐	☐	☐	☐

15. My friends think I should eat 5 portions of fruit and vegetable per day

Strongly agree				Strongly disagree
1	2	3	4	5
☐	☐	☐	☐	☐

16. My doctor and midwife think I should eat 5 portions of fruit and vegetable per day

Strongly agree				Strongly disagree
1	2	3	4	5
☐	☐	☐	☐	☐

17. Eating 5 portions of fruit and vegetable per day will reduce the chances of me getting -

Cancer — Yes ☐ No ☐ Don't know ☐

Heart disease — No ☐ Yes ☐ Don't know ☐

Diabetes — Don't Know ☐ Yes ☐ No ☐

Obesity — Yes ☐ Don't know ☐ No ☐

18. Eating 5 portions of fruit and vegetables per day while I am pregnant is good for my baby.

No ☐ Don't know ☐ Yes ☐

19. How would you rate your health today?

Very good — Very poor

1	2	3	4	5
☐	☐	☐	☐	☐

20. Have you been given any advice about your diet since you became pregnant?

Yes ☐ No ☐

Please specify the advice you were given ______________________________

____

____

21. Where do you find out information about diet and health?

Please tick all that you use

**Magazines**	
**TV programmes**	
**TV adverts**	
**Medical appointments/clinics**	
**Health promotion leaflets**	
**Friends**	
**Family**	
**Social media – like Facebook**	
**Other (please specify)**	

22. This part of the questionnaire is designed to look at how you see yourself as a person.

Please tick one box on each line that you think best describes you

I see myself as...	Disagree strongly 1	Disagree moderately 2	Disagree a little 3	Neither agree or disagree 4	Agree a little 5	Agree moderately 6	Agree strongly 7
Extraverted Enthusiastic							
Critical Quarrelsome							
Dependable Thorough							
Anxious Easily upset							
Open to new experience Complex							
Reserved Quiet							
Sympathetic Warm							
Disorganised Careless							
Calm Even-tempered							
Conventional Uncreative							
